# The Opioid System in Rainbow Trout Telencephalon Is Probably Involved in the Hedonic Regulation of Food Intake

**DOI:** 10.3389/fphys.2022.800218

**Published:** 2022-03-01

**Authors:** Adrián Díaz-Rúa, Mauro Chivite, Sara Comesaña, Marta Conde-Sieira, José L. Soengas

**Affiliations:** Centro de Investigación Mariña, Laboratorio de Fisioloxía Animal, Departamento de Bioloxía Funcional e Ciencias da Saúde, Facultade de Bioloxía, Universidade de Vigo, Vigo, Spain

**Keywords:** opioid, food intake, brain, palatable diet, nutritional condition

## Abstract

We hypothesize that opioids are involved in the regulation of food intake in fish through homeostatic and hedonic mechanisms. Therefore, we evaluated in rainbow trout (*Oncorhynchus mykiss*) hypothalamus and telencephalon changes in precursors, endogenous ligands and receptors of the opioid system under different situations aimed to induce changes in the homeostatic (through fasted/fed/refed fish) and hedonic (through feeding fish a control or a palatable high-fat diet) regulation of food intake. No major changes occurred in parameters assessed related with the nutritional condition of fish (fasted/fed/refed), allowing us to suggest that the opioid system seems not to have an important role in the homeostatic regulation of food intake in rainbow trout. The responses observed in telencephalon of rainbow trout fed the palatable high-fat diet included a decrease in mRNA abundance of the opioid precursor *penka*, in a way similar to that known in mammals, and increased mRNA abundance of the opioid receptors *oprd1* and *oprk1* supporting a role for telencephalic opioid system in the hedonic regulation of food intake in fish.

## Introduction

In mammals, opioids are analgesic and hypnotic drugs acting through delta- (DOP), kappa- (KOP), and mu-opioid (MOP) receptors encoded by the genes *Oprd1, Oprk1*, and *Oprm1*, respectively. Leu- (LEU) and Met-enkephalin (MET) are endogenous ligands of these receptors together with other opioids such as dynorphins, β-endorphin, and enkephalins produced by cleavage of the peptide precursors preprodynorphin (PDYN), pro-opiomelanocortin (POMC), prepronociceptin (PNOC), and preproenkephalin (PENK) (Bodnar, 2015). Opioids are involved in many different roles in mammals including cognition, nociception, cardiovascular function, gastrointestinal function, and immune response, among others (Bodnar, 2015). The opioid receptors are widely distributed throughout the CNS including brain areas involved in the regulation of food intake, such as hypothalamus and telencephalon (Bodnar, 2019). Accordingly, treatment with opioid antagonists (naltrexone or naloxone) reduced the intake of palatable food whereas treatment with agonists (encephalin analogs or morphine) increase food consumption (Cota et al., 2006; Nogueiras et al., 2012; Bodnar, 2019). In addition to the regulation of food intake by homeostatic signals, opioids are also involved in the hedonic aspects of food intake through reward mechanisms in mammals (Le Merrer et al., 2009; Berthoud et al., 2017). The hedonic regulation of food intake operates through sensorial and rewarding signals. They can prevail over homeostatic regulation resulting in overconsumption of palatable food even under conditions when energy requirements are covered (Berthoud and Morrison, 2008; Lutter and Nestler, 2009).

In fish, available evidence supports the presence of components of the opioid system (Vallarino et al., 2012; Larhammar et al., 2015; Herrero-Turrión, 2017; Demin et al., 2018; Bao et al., 2019). Most of this evidence relates to the presence, especially in brain areas, of opioid receptors as demonstrated through assessment of their mRNA abundance, as observed in zebrafish *Danio rerio* (López-Bellido et al., 2012; Jimenez-Gonzalez et al., 2016; Thörnqvist et al., 2019), goldfish *Carassius auratus* (Kobayashi et al., 2014) or rainbow trout *Oncorhynchus mykiss* (Rosengren et al., 2018). There is also information regarding the presence of the endogenous opioid ligands as in gilthead sea bream *Sparus aurata* (Albrizio et al., 2014) or their precursors as in spotted snakehead *Channa punctata* (Singh and Rai, 2009), pejerrey *Odontesthes bonariensis* (Vigliano et al., 2011), and goldfish (Liu et al., 2018). As for physiological roles of this system, opioids are apparently involved in comparable functions to those known in mammals as supported by results obtained in several fish species. These include: anti-nociception in rainbow trout (Jones et al., 2012; Gräns et al., 2014) and silver catfish *Rhamdia quelen* (Rodrigues et al., 2019), analgesia in goldfish (Kobayashi et al., 2014), addiction in zebrafish (López-Bellido et al., 2012; Jimenez-Gonzalez et al., 2016), stress response in rainbow trout (Rosengren et al., 2018) and gilthead sea bream (Albrizio et al., 2014), immune response in spotted snakehead (Singh and Rai, 2009), zebrafish (Liu et al., 2019) and northern pike *Esox lucius* (Bosi et al., 2020), reproduction in goldfish (Liu et al., 2018), oxidative stress in zebrafish (Liu et al., 2019), and aggressive/shy behavior in zebrafish (Thörnqvist et al., 2019). However, in contrast to mammals, there is almost no information in fish regarding the putative role of opioids in food intake regulation (Bao et al., 2019) with few studies demonstrating that β-endorphin stimulates food intake in goldfish (De Pedro et al., 1995) and tench *Tinca tinca* (Guijarro et al., 1999) apparently through MOP receptor (De Pedro et al., 1996).

The regulation of food intake in fish is dependent upon interaction of signals of homeostatic and hedonic nature (Delgado et al., 2017; Soengas et al., 2018). The homeostatic signals arise from the necessity of maintaining body energy, while hedonic signaling results from pleasure and/or sensory perception. Both types of signals are integrated in brain regions notably in hypothalamus and telencephalon (Delgado et al., 2017; Soengas, 2021). The knowledge about regulation of food intake in fish is mostly restricted to homeostatic mechanisms. Few available studies addressed hedonic mechanisms including our recent studies regarding the involvement of endocannabinoids (Díaz-Rúa et al., 2020a,b, 2021). The involvement of opioids in food intake regulation is a reasonable hypothesis based on findings achieved in several fish species. These include (1) the increased food intake observed after intracerebroventricular (ICV) administration of β-endorphin in goldfish (De Pedro et al., 1995) and tench (Guijarro et al., 1999); (2) the effects of naloxone (opioid antagonist) in food intake of goldfish (De Pedro et al., 2000); (3) the presence in hypothalamus and telencephalon of opioid receptors either considering binding sites (González-Núñez et al., 2006) or expression characterized through *in situ* hybridization (Sivalingam et al., 2020) and qPCR (Rosengren et al., 2018; Thörnqvist et al., 2019); (4) the expression of opioid precursors in hypothalamus and telencephalon, as observed in spotted snakehead (Singh and Rai, 2009), rainbow trout (Vecino et al., 1992), tilapia *Oreochromis mossambicus* (Vijayalaxmi et al., 2020), and goldfish (Liu et al., 2018).

We hypothesize that opioids are involved in food intake regulation in fish through homeostatic and hedonic mechanisms. Therefore, we evaluated in rainbow trout (*Oncorhynchus mykiss*) changes in precursors, endogenous ligands and receptors of the opioid system under different situations aimed to induce changes in the homeostatic and hedonic regulation of food intake. For homeostatic mechanisms, we evaluated periprandial (from 30 min before feeding until 180 min after feeding) changes under different feeding conditions such as fasting, feeding, and refeeding after fasting. For hedonic mechanisms, we evaluated the response of opioid system in fish fed a palatable high-fat diet known to induce a reward food intake response in this species (Díaz-Rúa et al., 2020a).

## Materials and Methods

### Fish

Rainbow trout coming from a local fish farm (A Estrada, Spain) were kept for 1 month in tanks of 100 L with a photoperiod of 12L:12D (lights on at 08:00 h, lights off at 20:00 h) in tap water previously dechlorinated and under a controlled temperature of 15°C. Once a day food were supplied to fish (at 11.00 h) until satiation with commercial aquafeed (proximate food composition: 44% crude protein, 2.5% carbohydrates, 21% crude fat, and 17% ash; 20.2 MJ/kg of feed; Biomar, Dueñas, Spain). The experiments performed are in agreement with the directions of the Council of the European Union (2010/63/UE) and the Spanish Government (RD 53/2013) for the use of experimental animals as approved by the Ethics Committee of the Universidade de Vigo.

### Experimental Design

#### Experiment 1: Effects of Energy Status on Opioid System

Twenty four or seventy two hours before experiment, food was not provided to rainbow trout (80 ± 3 g). On the day of experiment, food was supplied to fish previously fasted for 24 h (fed group) or not supplied (fasted group). The fish that were fasted for 72 h were refed on the day of the experiment (refed group). Then, at different pre- and postprandial times (−30 min, 0 min, + 30 min and + 180 min), fish were softly anesthetized by adding 2 phenoxyethanol (Sigma, 0.02% v/v) directly in the tank, netted and sacrificed by decapitation. Hypothalamus and telencephalon were dissected corresponding to *N* = 10 fish per feeding condition (fasted, fed and refed) and sampling time. The samples obtained were immediately frozen and stored at −80°C for future determination of opioid levels (*N* = 10 fish per experimental group), or assessment of mRNA levels by qRT-PCR (*N* = 6 fish per experimental group).

#### Experiment 2: Effects of Dietary Fat Content on Opioid System

The two experimental diets were formulated and manufactured by Sparos (Portugal) containing the same protein level (38–40% of dry matter) but different crude fat levels: A moderate fat content (13%) in the case of control diet (CD) which is comparable to commercial aquafeeds, or elevated crude fat levels (35%) in the case of the high-fat diet (HFD). The formulation and theoretical composition of the experimental diets is included as Supplementary Table 1. These diets are the same than those previously described in our previous study (Díaz-Rúa et al., 2020a) where it was shown that HFD induce a reward food intake response in rainbow trout as suggested by the preference of fish for this diet (despite it higher energy content compared to CD) and by the changes observed in parameters related with the cannabinoid system. After an acclimation period of 1 month, rainbow trout (125.4 ± 0.9 g) were haphazardly distributed into 6 different experimental tanks (10 fish/100 L tank) and provided with commercial diet until satiation once a day during 7 days. After this period, fish were fed until satiation with the experimental diets (CD and HFD) once daily during 4 days, as in this period time it was previously demonstrated a higher intake of HFD compared with a control diet (CD) (Díaz-Rúa et al., 2020a). The CD was supplied to 3 tanks whereas the other 3 tanks were fed with HFD. On the experimental day, fish were fed the experimental diets (CD or HFD) at 11 h and sampled 3 and 6 h later. For sampling, 2 phenoxyethanol (Sigma, 0.02% v/v) was directly added in the tank and the anesthetized fish were removed, weighted, and then sacrificed by decapitation. We dissected *N* = 10 fish per diet on each sampling time for obtaining hypothalamus and telencephalon immediately frozen in dry ice and stored at −80°C. Six fish (out of 10) per treatment at each sampling time were used for the assessment of mRNA levels by qRT-PCR.

### mRNA Abundance Analysis by Real-Time Quantitative RT-PCR

Total RNA was obtained from the removed tissues (approx. 20 mg) by following the Trizol reagent commercial protocol (Life Technologies, Grand Island, NY, United States). Then, RNA samples were treated with RQ1-DNAse (Promega, Madison, WI, United States) for removing any possible DNA contamination. Two μg of each sample of total RNA were converted to cDNA by performing reverse transcription with Superscript II reverse transcriptase (Life Technologies) and random hexaprimers (Invitrogen). It was assessed the gene expression levels by real-time quantitative RT-PCR (q-PCR) performed with the iCycler iQ™ (BIO-RAD, Hercules, CA, United States). For that purpose, it was used 1 μL of each cDNA sample, adding the MAXIMA SYBR Green qPCR Mastermix (Life Technologies) and 50–500 nM of each specific primer, obtaining a total PCR reaction volume of 15 μL. Then, we assessed mRNA abundance of transcripts related to (1) opioid receptors such as opioid receptor delta 1 (*oprd1*), opioid receptor kappa 1 (*oprk1*), opioid receptor mu 1 (*oprm1*), opioid growth factor-1 (*ogf1*), and nociceptin opioid peptide receptor (*norp*); (2) opioid precursors such as prodynorphin (*pdyn*) and proenkephalin a (*penka*). In Table 1 are presented the sequences of the forward and reverse primers used for each transcript expression which were designed based on sequences available in GenBank. Thermal cycling protocol consisted in an initial step of incubation at 95°C for 10 min for activation of the hot-start iTaq DNA polymerase and then 35 cycles consisting of heating at 95°C for 20 s and an annealing and extension step at 60°C for 20 s. Following the final PCR protocol, the melting curve (55°C temperature gradient at 0.5°C/s from 55 to 94°C) was obtained to ensure the primer specificity with only one fragment amplified. Negative controls were performed for each reaction by adding samples without reverse transcriptase and samples without template. Efficiency values between 85 and 100% were obtained (The R^2^ for all genes assessed was higher than 0.985). For the relative quantification of the target gene transcript it was used β-actin (*actb*) and elongation factor 1-α (*eef1a)* as housekeeping genes which were stably expressed in this experiment. Calculations were made by following the Pfaffl (2001).

**TABLE 1:** Nucleotide sequences of the PCR primers used to evaluate mRNA abundance by RT-qPCR.

Gene	Forward primer	Reverse primer	Accession number	Annealing T (°C)
*actb*	GATGGGCCAGAAAGACAGCTA	TCGTCCCAGTTGGTGACGAT	NM_001124235.1	60
*eef1a1*	GGGCAAGGGCTCTTTCAAGT	CGCAATCAGCCTGAGAGGT	AF498320	59
*norp*	GGAACGCAGGCAACAACCCA	GGTCTCGTTGAACCCACCGC	XM_036989001.1	60
*penka*	GCGACTTGGTAAGCACCGGG	GCGCACAGTCCTTCTCGCAG	XM_021613078.2	60
*pdyn*	ATGCGCGCAACAAATCCTCA	TCTCTGGCGTGGACCGTGG	XM_021615876.2	60
*ogf1*	CCGGACGATTTAGTGTGCGT	CCTTGGCAGCCCTCATGTTCC	XM_021615595.2	60
*oprd1*	AGAGGATCCCATGTCCCCAAT	GCCAGAGCCAGGTTGAAGATG	XM_021562945.2	60
*oprk1*	CATCTTCGGCTTCGTGGCCC	GGGGGCGCCAGCATCTTAAC	XM_036986795.1	60
*oprm1*	CATGCGCACGCTCTTCAGGC	TCTGCCTGTCATGTTCACCGC	XM_021581332.2	60

*actb, β-actin; eef1a1, elongation factor 1α; norp, nociceptin receptor-like; penka, proenkephalin a; pdyn, prodynorphin; ogf1, opioid growth factor receptor; oprd1, delta-type opioid receptor; oprk1, kappa-type opioid receptor 1; oprm1, mu-type opioid receptor.*

### Determination of the Levels of Met-Enkephalin and LEU by LC-MS/MS

The levels of opioids Leu-enkephalin (LEU) or Met-enkephalin (MET) were quantified in hypothalamus through LC-MS/MS in Centro de Apoio Científico-Tecnolóxico (CACTI) of Universidade de Vigo. The system used was an Infinity liquid 1,260 chromatographic system (HPLC-MS/MS), composed by an autosampler, a column thermostat, and a binary solvent management system coupled to a triple quadrupole (Agilent Technologies 6430) and equipped with an Atmospheric Pressure Chemical Ionization ion source (APCI). The analytes were separated using a reversed phase Kinetex C18 XB 50 × 2.10 mm from Phenomenex (Torrance, CA, United States), packed with 2 μm average diameter core-shell particles. The mobile phases used were water (phase A) and 2.5 mM formic acid in methanol (phase B), at a flow rate of 0.4 mL min^–1^. The gradient elution scheme used consist in a decrease of the organic phase from 45 to 5% in 4 min that remained stable until 5.5 min. Then, after 1.5 min of 5% A, the column returned to the original ratio of 55% B and 45% A within 3.5 min to enable equilibration of the column, resulting in a total run-time of 9 min. All analytes were detected in positive ionization with a capillary voltage of 4,800 V, nebulizer gas (air) at 60 psi and the turbo gas (nitrogen) was at 30 psi and 600°C. All source and instrument parameters for the analytes assessed were tuned by injecting standard solutions at a concentration of 100 ng mL^–1^ (containing 10 mM formic acid) by a syringe pump at 10 μL min^–1^. Peak areas for the selected ions were determined using PE Sciex package Multiview 1.4 and the internal standard method was performed by quantification. The standards used for the determination of opioids were Leucine Enkephalin acetate salt hydrate (Leu-enkephalin) and [Met5] Enkephalin acetate salt hydrate (Met-enkephalin) both purchased at Sigma–Aldrich (Munich, Germany).

### Statistics

The normality and homogeneity of variance of data were previously checked and data was log-transformed when needed. In experiment 1, comparisons among groups were carried out using two-way ANOVA with feeding condition (fasted, fed or refed) and periprandial time (−30, 0, + 30, + 180 min) as main factors. In experiment 2, comparisons among groups were performed using two-way ANOVA with treatment (CD or HFD) and time (3 or 6 h) as main factors. In both experiments where a significant effect was noticed, *post hoc* comparisons were carried out by a Student-Newman-Keuls test, and differences were considered statistically significant at *p* < 0.05. All analyses were performed using SigmaPlot version 12.0 (Systat Software Inc., San Jose, CA, United States).

## Results

### Experiment 1: Effects of Energy Status on Opioid System

Table 2 displays *p*-values of effects of main factors periprandial time and feeding condition and their interaction. A significant interaction occurred for mRNA abundance of *oprd1* (F6 = 2.527, *p* = 0.033) and *penka* (F6 = 2.493, *p* = 0.037) in hypothalamus.

**TABLE 2:** *p*-values obtained after two-way analysis of variance in experiment 1 of levels of opioids in hypothalamus, and mRNA abundance of transcripts related to the opioid system in hypothalamus and telencephalon of rainbow trout.

	Hypothalamus			Telencephalon		
Parameter	F	T	FxT	F	T	FxT
MET	0.674	0.770	0.595			
LEU	0.072	0.225	0.321			
*oprd1*	0.837	0.005	0.033	0.697	0.143	0.923
*oprk1*	0.585	0.852	0.996	0.385	0.179	0.907
*oprm1*	0.321	0.120	0.103	0.922	0.425	0.544
*ogf1*	0.231	0.175	0.515	0.781	<0.001	0.055
*norp*	0.774	0.970	0.426	0.010	0.151	0.990
*penka*	0.896	0.338	0.037	0.149	0.415	0.792
*pdyn*	0.378	0.209	0.359	0.041	0.440	0.507

*Feeding condition (F) and periprandial time (T) were the main factors, and Feeding × Time (F × T) is the first order interaction.*

**FIGURE 1:**
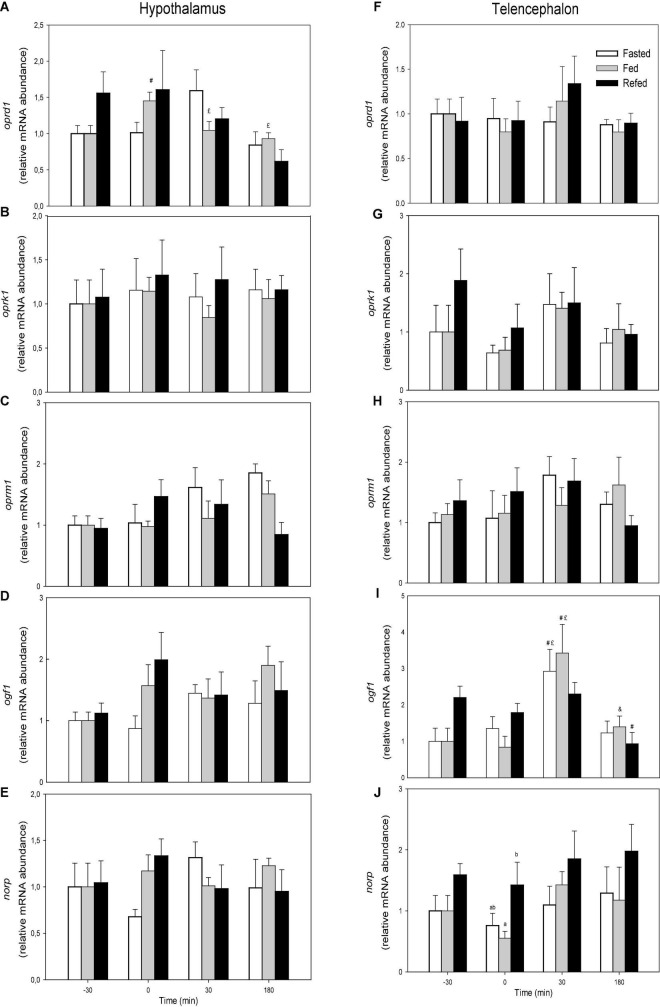
mRNA abundance of *oprd1* **(A,F)**, *oprk1* **(B,G)**, *oprm1* **(C,H)**, *ogf1* **(D,I)**, and *norp* **(E,J)** in hypothalamus **(A–E)** and telencephalon **(F–J)** of fasted, fed an refed rainbow trout sampled at different periprandial times (−30, 0, 30, 180 min). Each value is the mean + S.E.M. of 6 fish. Different letters indicate significant differences (*P* < 0.05) among feeding condition within each sampling time. ^#^Significantly different from T = −30 at the same treatment; ^£^significantly different from T = 0 at the same treatment; ^&^significantly different from T = 30 at the same treatment.

Changes in the mRNA abundance of opioid receptors are displayed in Figure 1. The values of *oprd1* did not display changes when comparing feeding conditions whereas values in fasted group in hypothalamus decreased with time from 0 to 30 min (F3 = 4.936, *p* = 0.005) (Figure 1A). The values of *ogf1* in telencephalon (Figure 1I) of fasted and fed groups after 30 min were higher than those at times −30 and 0 within the same treatments whereas the value after 180 min in refed fish was lower than the same treatment at time −30 (F3 = 12.366, *p* < 0.001). The mRNA abundance of *norp* in telencephalon (Figure 1J) was higher in refed fish than in fed fish at time 0. No significant changes occurred in mRNA abundance of the other transcripts evaluated.

**FIGURE 2:**
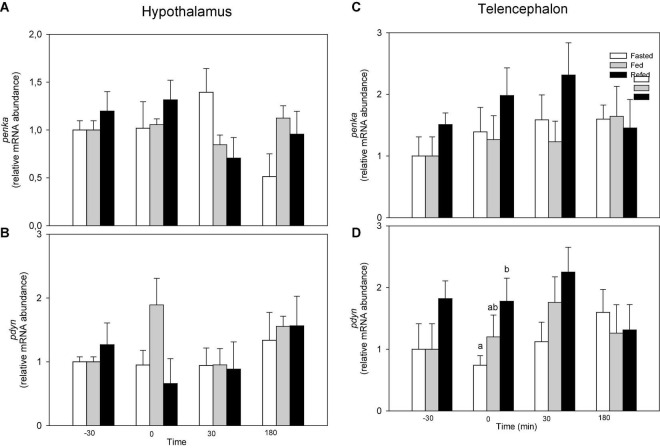
mRNA abundance of *penka* **(A,C)** and *pdyn* **(B,D)** in hypothalamus **(A,B)** and telencephalon **(C,D)** of fasted, fed an refed rainbow trout sampled at different periprandial times (−30, 0, 30, 180 min). Each value is the mean + S.E.M. of 6 fish. Different letters indicate significant differences (*P* < 0.05) among feeding condition within each sampling time.

Changes in mRNA abundance of opioid precursors is shown in Figure 2. No changes occurred in hypothalamus (Figures 2A,B). In telencephalon, no changes occurred in mRNA abundance of *penka* (Figure 2C) while the value of *pdyn* mRNA abundance in refed fish was higher than that of fasted fish at time 0 of *pdyn* mRNA abundance (F2 = 3.435, *p* = 0.041) (Figure 2D).

**FIGURE 3:**
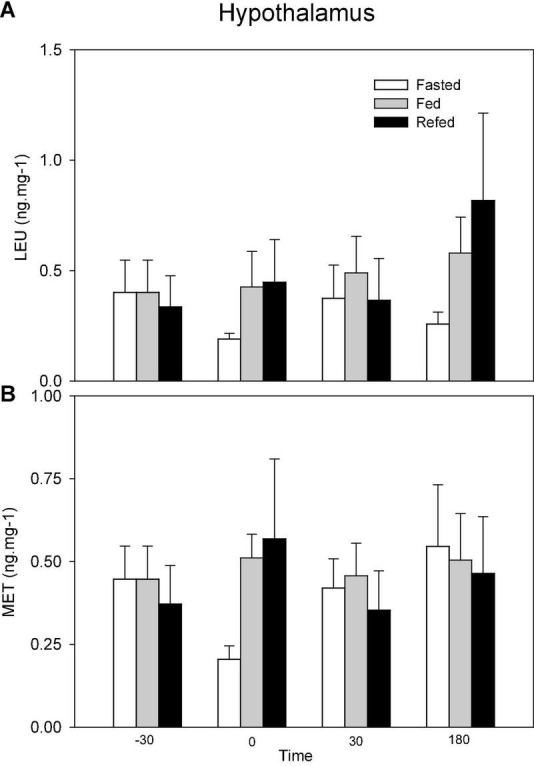
Levels of Leu-enkephalin (LEU, **A**) and Met-enkephalin (MET, **B**) in hypothalamus of fasted, fed an refed rainbow trout sampled at different periprandial times (-30, 0, 30, 180 min). Each value is the mean + S.E.M. of 10 fish.

Changes in the levels of opioids in hypothalamus are shown in Figure 3. No significant changes occurred.

### Experiment 2: Effects of Dietary Fat Content on Opioid System

Table 3 displays *p*-values of effects of main factors time and dietary condition and their interaction. A significant interaction occurred in mRNA abundance of *oprd1* (F1 = 5.448, *p* = 0.030), *oprk1* (F1 = 8.134, *p* = 0.010) and *oprm1* (F1 = 3.635, *p* = 0.018) in hypothalamus and *oprd1* (F1 = 9.054, *p* = 0.008) and *oprk1* (F1 = 5.229, *p* = 0.034) in telencephalon.

**TABLE 3:** *p*-values obtained after two-way analysis of variance in experiment 2 of and mRNA abundance of transcripts related to the opioid system in hypothalamus and telencephalon of rainbow trout.

	Hypothalamus			Telencephalon		
Parameter	D	T	DxT	D	T	DxT
*oprd1*	0.950	0.901	0.030	0.035	0.177	0.008
*oprk1*	0.047	0.189	0.010	0.048	0.532	0.034
*oprm1*	0.700	0.583	0.018	0.112	0.864	0.503
*ogf1*	0.818	0.540	0.071	0.542	0.458	0.851
*norp*	0.478	0.159	0.136	0.043	0.299	0.170
*penka*	0.944	0.380	0.299	0.005	0.148	0.389
*pdyn*	0.136	0.298	0.153	0.105	0.410	0.439

*Dietary condition (D) and time (T) were the main factors, and dietary condition × Time (D × T) is the first order interaction.*

**FIGURE 4:**
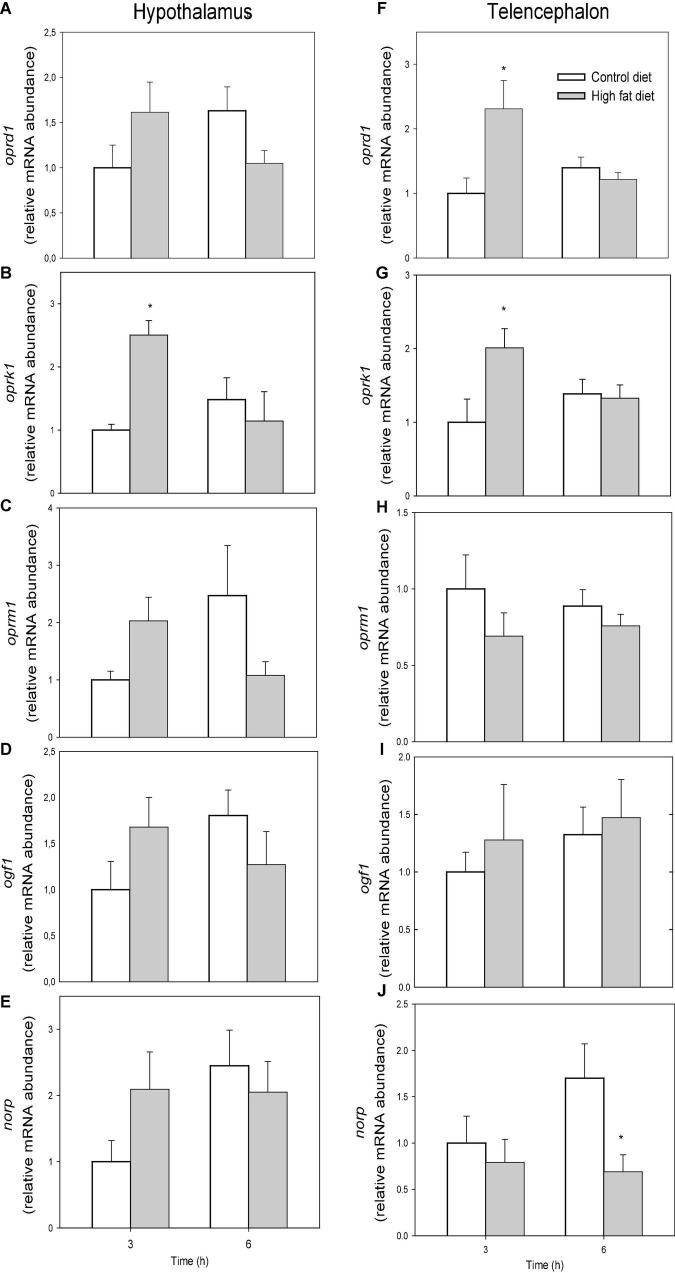
mRNA abundance of *oprd1* **(A,F)**, *oprk1* **(B,G)**, *oprm1* **(C,H)**, *ogf1* **(D,I)**, and *norp* **(E,J)** in hypothalamus **(A–E)** and telencephalon **(F–J)** of rainbow trout fed with control diet (CD) or high-fat diet (HFD) sampled at different postfeeding times (3 and 6 h). Each value is the mean + S.E.M. of 6 fish. * Significantly different (*P* < 0.05) from fish fed with CD at the same time.

The mRNA abundance of opioid receptors is shown in Figure 4. The mRNA abundance of *oprk1* was higher in fish fed HFD than in fish fed control diet after 3 h in hypothalamus (F1 = 3.256, *p* = 0.047) (Figure 4B) and telencephalon (F1 = 4.157, *p* = 0.048) (Figure 4G). The values of *oprd1* in telencephalon (Figure 4F) of fish fed HFD were higher after 3 h than in fish fed control diet (F1 = 5.203, *p* = 0.035). No changes occurred in mRNA abundance of *oprm1* (Figures 4C,H) and *ogf1* (Figures 4D,I). The values of *norp* in telencephalon (Figure 4J) after 6 h were lower in fish fed HFD than those fed control diet (F1 = 4.727, *p* = 0.043).

**FIGURE 5:**
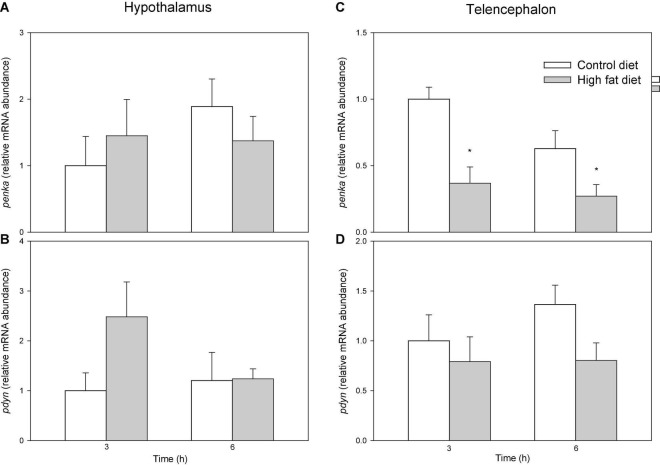
mRNA abundance of *penka* **(A,C)**, and *pdyn* **(B,D)** in hypothalamus **(A,B)** and telencephalon **(C,D)** off of rainbow trout fed with control diet (CD) or high-fat diet (HFD) sampled at different postfeeding times (3 and 6 h). Each value is the mean + S.E.M. of 6 fish. *Significantly different (*P* < 0.05) from fish fed with CD at the same time.

Figure 5 shows mRNA abundance of opioid precursors. In telencephalon values were lower in fish fed HFD than in fish fed control diet after 3 and 6 h for *penka* (F1 = 10.083, *p* = 0.005) (Figure 5C) but not *pdyn* (Figure 5D) while no changes occurred in hypothalamus (Figures 5A,B).

## Discussion

This is the first study, as far as we are aware, assessing in a teleost fish the simultaneous presence of the main components of the opioid system in brain areas involved in food intake regulation like hypothalamus and telencephalon. Available studies focus only on the presence in those areas of specific components such as receptors or precursors (Demin et al., 2018; Bao et al., 2019) or to the effects of β-endorphin on food intake (De Pedro et al., 1995, 1996; Guijarro et al., 1999). If opioids are involved in homeostatic regulation of food intake, their parameters should respond to changes in the nutritional condition of fish conditioned by the presence/absence of food as previously demonstrated for many other endocrine systems in fish (Soengas et al., 2018). However, in hypothalamus the nutritional condition of fish (fasted, fed, refed) did not alter levels of opioids, and mRNA abundance of precursors and receptors (except for *oprd1*) allowing us to suggest the absence of a relevant role for opioid system in this area in homeostatic regulation of food intake. In telencephalon the mRNA abundance of the *norp* receptor and the *pdyn* precursor increased in refed fish when compared with fasted fish at time 0 also suggesting a marginal role in the regulation in food intake related to energetic conditions of this brain area. This is in contrast to that known in mammalian models (Nogueiras et al., 2012) where different homeostatic conditions alter the levels and expression of opioid peptides, their precursors and receptors (Bodnar, 2019). Thus, starvation increases whereas obesity decreases gene expression of MOP in rat (Barnes et al., 2008; Robinson et al., 2015). Food deprivation also decreases *norp* mRNA levels and modifies the activity of KOP in several areas of rat brain (Wolinsky et al., 1994; Przydzial et al., 2010). Furthermore, mRNA levels of β-endorphin, prodynorphin and immunoreactivity of L-enkephalin decrease in mammals under chronic food restriction (Bodnar, 2019). In the present study, we observed lower *pdyn* mRNA levels in fasted than refed fish and a similar trend was observed (although not significant) for Met and Leu-enkephalin levels in hypothalamus at feeding time.

The opioid system in mammals is involved in the hedonic regulation of food intake (Berthoud et al., 2017). If opioids are involved in food palatability, the opioid system should respond to the intake of palatable food (Cota et al., 2006). To assess such possibility in fish, we evaluated the response of the components of this system to the presence of a highly palatable food known to induce in rainbow trout an increase in food intake together with the activation of another system involved in the hedonic regulation of food intake, such as the endocannabinoid system (Díaz-Rúa et al., 2020a). In hypothalamus, feeding the palatable HFD resulted in a single change, particularly the increased mRNA abundance of the receptor *oprk1* after 3 h. The reward response mediated by other systems such as endocannabinoids also involves changes in hypothalamus (Díaz-Rúa et al., 2020a). It seems therefore that the involvement of the opioid system in the reward response in rainbow trout is restricted to brain areas other than hypothalamus. In this way, telencephalon is suggested to be the main brain area involved in hedonic regulation of food intake in fish (Mueller and Wullimann, 2009). Accordingly, dense networks of enkephalinergic fibers are present in the telencephalon of different fish species (Vallarino et al., 2012) including rainbow trout (Vecino et al., 1992) supporting the importance of the opioid system in this brain area. Enkephalins, derived from the precursors PDYNs and PENKs are the most relevant opioid peptides (Herrero-Turrión, 2017). Not surprisingly, in mammals a highly palatable food induces a decrease in enkephalin levels in hypothalamus (Kelley et al., 2003) and prodynorphin levels in cortex (Blasio et al., 2014; Murray et al., 2014). In the present study, we observed changes in telencephalon of fish fed HFD including decreased mRNA abundance of the opioid precursor *penka*, i.e., a response comparable to that known in other brain regions in mammalian brain. However, many other studies in mammals report increased enkephalin levels after increased intake of dietary fats (Chang et al., 2010; Bodnar, 2019). These differences could relate to the duration of administration and accessibility of palatable food since a repeated consumption of high palatable food could induce neuroadaptive responses (Kelley et al., 2003).

Furthermore, we also observed that mRNA abundance of opioid receptors *oprd1* and *oprk1* increased after 3 h of feeding with HFD. In mammals an increase occurs in MOP binding in cortex, amygdala and hippocampus (Murray et al., 2014) and ICV treatment with MOP agonist results in increased intake of highly palatable food (Murray et al., 2014; Bodnar, 2019). The pharmacological properties of opioid receptors in zebrafish are similar to those of their mammalian counterparts (Bao et al., 2019). Considering that in the present study *oprm1* mRNA abundance did not display changes we may suggest that, in contrast to mammals, DOP and KOP but not MOP would be involved in hedonic regulation of food intake in rainbow trout telencephalon. However, De Pedro et al. (1996) demonstrated that intracerebroventricular treatment with MOP antagonists in goldfish, but not with DOP and KOP antagonists, counteracts the increase in food intake elicited by β-endorphin treatment suggesting that the involvement of different opioid receptors might be species-dependent. In zebrafish, MOP and DOP agonists produce a reward response whereas KOP agonists elicit an aversion response (Demin et al., 2018). This response is comparable to that known in mice where ICV injection of receptor agonist differs in the response of feeding a HFD in a way that DOP agonist reduces food intake while MOP agonist increases food intake (Kaneko et al., 2014). However, we observed a parallel response of the mRNA abundance of *oprm1* and *oprk1* in telencephalon in response to feeding a HFD suggesting that the differential role between both receptors suggested for zebrafish is not apparent in rainbow trout. This is interesting since in zebrafish again it seems that the main effects of opioids in processes like analgesia, tolerance and addiction appear to be mediated by MOP (López-Bellido et al., 2012; Arévalo et al., 2018). Since no other studies evaluated in zebrafish changes in the mRNA abundance of these receptors related to food intake, we are not sure if changes described in rainbow trout are species-specific or teleost-specific. The behavior of the *norp* receptor was the opposite with decreased mRNA abundance in telencephalon of fish fed HFD. The few studies underlying the role of NORP receptor on food intake regulation show decreased expression of this receptor in rats under food deprivation (Przydzial et al., 2010; Ren et al., 2013) and a lack of response on the intake of palatable diets after central administration of its ligand, the nociceptin/orphanin FQ peptide (Bodnar, 2019). These results suggest a role of *norp* different from the other opioid receptors showing hyperphagic properties that are more related with the inhibition of anorexigenic pathways than the activation of the orexigenic ones (Olszewski and Levine, 2004; Bodnar, 2019). In any case, this is the first time in which changes in the mRNA abundance of opioid receptors are reported under situations eliciting changes in food intake since previous studies in fish relate these changes to other processes such as development (Sanchez-Simon and Rodriguez, 2008).

We are therefore suggesting that activation of opioid system in rainbow trout telencephalon is involved in the reward increase in food intake of a palatable (HFD) diet. As for downstream mechanisms involved, we might suggest a putative role for cAMP response element binding protein (CREB). In mice brain regions acute administration of MOP agonists led to increased CREB phosphorylation while CREB phosphorylation decrease is known to occur under inhibition of food intake (Ren et al., 2013). In fish, CREB is suggested to be involved in MOP downstream mechanisms in zebrafish (Jimenez-Gonzalez et al., 2016). Considering that CREB phosphorylation status usually decrease in fish brain under conditions eliciting a decrease in food intake (Soengas, 2021), we could suggest that an increased CREB phosphorylation is involved in opioid mechanisms in telencephalon of rainbow trout.

In summary, we evaluated in rainbow trout hypothalamus and telencephalon changes in precursors, endogenous ligands and receptors of the opioid system under different situations aimed to induce changes in the homeostatic (through different nutritional conditions induced by the presence/absence of food) and hedonic (through feeding fish a control or a HFD palatable diet) regulation of food intake. Since no major changes occurred in parameters assessed in the presence/absence of food, we suggest, in contrast to the mammalian model, that the opioid system appear not to have an important role in the homeostatic regulation of food intake in rainbow trout. The responses observed in telencephalon of rainbow trout fed the HFD included a decrease in mRNA abundance of the opioid precursor *penka*, in a way similar to that known in mammals, and increased mRNA abundance of the opioid receptors *oprd1* and *oprk1* supporting a role for DOP and KOP receptors in the hedonic regulation of food intake in fish. These changes in receptors are different to those known in mammals where MOP is mainly involved in food intake regulation. Further studies including intracerebroventricular administration of opioids and receptor antagonists are necessary to elucidate the involvement of specific receptors in food intake regulation in rainbow trout.

## Data Availability Statement

The original contributions presented in the study are included in the article/Supplementary Material, further inquiries can be directed to the corresponding author/s.

## Ethics Statement

The animal study was reviewed and approved by the Ethics Committee of University of Vigo.

## Author Contributions

MC-S and JS conceived and designed the research. AD-R, MC, SC, and MC-S performed the experiments. AD-R and MC analyzed the data. AD-R, JS, and MC-S prepared figures. All authors drafted, edited, and revised manuscript, with MC-S and JS having the main contribution and approving the final version of manuscript. All authors interpreted results of the experiments.

## Conflict of Interest

The authors declare that the research was conducted in the absence of any commercial or financial relationships that could be construed as a potential conflict of interest.

## Publisher’s Note

All claims expressed in this article are solely those of the authors and do not necessarily represent those of their affiliated organizations, or those of the publisher, the editors and the reviewers. Any product that may be evaluated in this article, or claim that may be made by its manufacturer, is not guaranteed or endorsed by the publisher.
