# Supplementary material for: The Opioid System in Rainbow Trout Telencephalon Is Probably Involved in the Hedonic Regulation of Food Intake
Source: Front Physiol. 2022 Mar 1;13:800218. doi: 10.3389/fphys.2022.800218 (PMC8921556; doi:10.3389/fphys.2022.800218)
Supplement: Supplementary file 1 [file Table_1.docx]

| **Supplementary Table 1**. Ingredients and theoretical composition of the control diet (CD) and high-fat diet (HFD) used to fed rainbow trout | | |
| --- | --- | --- |
|  | Dietary treatment | |
|  | CD | HFD |
| *Feed ingredients (%)* |  |  |
| Fishmeal 70 LT ^1^ | 32.10 | 7.50 |
| CPSP G ^2^ | 2.50 | 7.00 |
| Wheat gluten | 8.00 | 26.80 |
| Soybean meal | 10.00 | 2.00 |
| Rapeseed meal | 7.00 | 0.00 |
| Wheat meal | 5.00 | 0.00 |
| Aquatex 8071 (gel) ^3^ | 17.00 | 14.00 |
| Fish oil | 9.00 | 34.00 |
| Vit & Min Premix ^4^ | 1.00 | 1.00 |
| Betaine | 0.50 | 0.50 |
| Binder (guar gum) ^5^ | 2.00 | 2.00 |
| Binder (Kieselghur) ^6^ | 1.00 | 1.00 |
| Anti-OX (Paramega PX Dry) | 0.50 | 0.50 |
| MCP ^7^ | 0.00 | 1.50 |
| Cellulose | 3.50 | 0.00 |
| L-Lysine | 0.40 | 1.40 |
| DL-Methionine | 0.30 | 0.30 |
| L-Threonine | 0.20 | 0.50 |
| *Theoretical composition* |  |  |
| Crude protein (% DM ^8^) | 39.96 | 38.38 |
| Crude fat (% DM) | 13.04 | 35.12 |
| Fiber (% DM) | 1.63 | 0.34 |
| Starch (% DM) | 12.54 | 9.04 |
| Gross Energy (KJ g^-1^ DM) | 17.28 | 24.43 |
| ^1^ Danish fishmeal LT70: 710 g crude protein (CP), 110 g crude fat (CF) per Kg fishmeal.  ^2^ CPSP: Soluble fish protein concentrate.  ^3^ Aquatex 8071: 23.5% crude protein, 1.0% crude fat.  ^4^ Premix for marine fish: Vitamins (IU or mg kg-1 diet): DL-alpha tocopherol acetate, 100 mg; sodium menadione bisulphate, 25 mg; retinyl acetate, 20000 IU; DL-cholecalciferol, 2000 IU; thiamin, 30 mg; riboflavin, 30 mg; pyridoxine, 20 mg; cyanocobalamin, 0.1 mg; nicotinic acid, 200 mg; folic acid, 15 mg; ascorbic acid, 1000 mg; inositol, 500 mg; biotin, 3 mg; calcium panthotenate, 100 mg; choline chloride, 1000 mg, betaine, 500 mg. Minerals (g or mg kg-1 diet): cobalt carbonate, 0.65 mg; copper sulphate, 9 mg; ferric sulphate, 6 mg; potassium iodide, 0.5 mg; manganese oxide, 9.6 mg; sodium selenite, 0.01 mg; zinc sulphate,7.5 mg; sodium chloride, 400 mg; calcium carbonate, 1.86 g; excipient wheat middlings.  ^5^ Guar gum HV109.  ^6^ Kieselghur (natural zeolite).  ^7^ MCP: Monobasic calcium phosphate: 18 % phosphorus, 23 % calcium.  ^8^ DM: Dry matter. | | |
